# The Genome of the Fungal-Interactive Soil Bacterium *Burkholderia terrae* BS001—A Plethora of Outstanding Interactive Capabilities Unveiled

**DOI:** 10.1093/gbe/evu126

**Published:** 2014-06-12

**Authors:** Irshad Ul Haq, Katharina Graupner, Rashid Nazir, Jan Dirk van Elsas

**Affiliations:** ^1^Department of Microbial Ecology, Center for Ecological and Evolutionary Studies (CEES), University of Groningen, The Netherlands; ^2^Department of Biomolecular Chemistry, Leibniz Institute for Natural Product Research and Infection Biology, Hans Knöll Institute, Jena, Germany; ^3^Department of Environmental Sciences, COMSATS Institute of Information Technology, Abbottabad, Pakistan

**Keywords:** *Burkholderia*, synteny, pan–core genome, regions of genomic plasticity, secretion systems, membrane transporters

## Abstract

*Burkholderia terrae* strain BS001, obtained as an inhabitant of the mycosphere of *Laccaria proxima* (a close relative of *Lyophyllum* sp. strain Karsten), actively interacts with *Lyophyllum* sp. strain Karsten. We here summarize the remarkable ecological behavior of *B. terrae* BS001 in the mycosphere and add key data to this. Moreover, we extensively analyze the approximately 11.5-Mb five-replicon genome of *B. terrae* BS001 and highlight its remarkable features. Seventy-nine regions of genomic plasticity (RGP), that is, 16.48% of the total genome size, were found. One 70.42-kb RGP, RGP76, revealed a typical conjugal element structure, including a full type 4 secretion system. Comparative analyses across 24 related *Burkholderia* genomes revealed that 95.66% of the total BS001 genome belongs to the variable part, whereas the remaining 4.34% constitutes the core genome. Genes for biofilm formation and several secretion systems, under which a type 3 secretion system (T3SS), were found, which is consistent with the hypothesis that T3SSs play a role in the interaction with *Lyophyllum* sp. strain Karsten. The high number of predicted metabolic pathways and membrane transporters suggested that strain BS001 can take up and utilize a range of sugars, amino acids and organic acids. In particular, a unique glycerol uptake system was found. The BS001 genome further contains genetic systems for the degradation of complex organic compounds. Moreover, gene clusters encoding nonribosomal peptide synthetases (NRPS) and hybrid polyketide synthases/NRPS were found, highlighting the potential role of secondary metabolites in the ecology of strain BS001. The patchwork of genetic features observed in the genome is consistent with the notion that 1) horizontal gene transfer is a main driver of *B. terrae* BS001 adaptation and 2) the organism is very flexible in its ecological behavior in soil.

## Introduction

As a result of the spatial separation in soil between bacterial cells and substrate, as well as the general recalcitrancy of substrate to degradation, soil bacteria most of the time perceive conditions of nutrient scarcity in their habitat and occur in a state of dormancy. However, there are so-called hot spots in soil in which soil bacteria are activated. One such hotspot for bacterial activity is the mycosphere, that is, the narrow zone of influence around the fungal hyphae in soil.

Evolutionary and genome-shaping events in soil bacteria (mutations and horizontal gene transfer and selective processes) are thought to mostly take place in high-activity microenvironments, and hence a thorough investigation of the genomic diversity of organisms dwelling in the mycosphere is warranted. On the basis of two different experimental setups, *Burkholderia terrae* like organisms were previously found to be key associates with fungal hyphae in soil (Warmink and van Elsas 2009). Indeed, since this initial discovery, a fascinating interaction between the mycosphere-isolated *B. terrae* strain BS001 and *Lyophyllum* sp. strain Karsten has been unveiled (Warmink and van Elsas 2009; Nazir, Zhang, et al. 2012).

The draft genome sequence of strain BS001 was recently announced (Nazir, Hansen, et al. 2012), but no detail on key genetic systems and on how the genomic information determines the ecophysiological behavior of the organism was provided. From the previous work, circumstantial ecologically based evidence was obtained for the contention that the presence of type 3 secretion system (T3SS), motility and biofilm formation traits as well as glycerol uptake and metabolism genes might offer selective advantages to the organism in the mycosphere. However, a tight linkage of the ecological data with genome features of strain BS001 has been lacking so far, in spite of the fact that this linkage is needed to build hypotheses that are testable in further experiments. Thus, in this study, we provide an in-depth analysis of the genome of *B. terrae* BS001, focusing on the plethora of genetic systems that potentially drive the interactions between *B. terrae* BS001 and the soil fungi it associates with. As the reference fungus, *Lyophyllum* sp. strain Karsten was used. To achieve our aim of assessing the genome in a broad sense and also addressing the uniqueness of particular features for strain BS001, we then performed comparative analyses of selected salient features of the genome of *B. terrae* BS001 with those of other (related) *Burkholderia* strains. From the data, we make inferences about the implications for the behavior of strain BS001 in soil, in an attempt to narrow the gap between (selected) genotype and phenotype.

## Results and Discussion

### Genome Properties

We first briefly describe the overall genome characteristics, before considering particular genetic systems that might relate to the ecological behavior of *B. terrae* BS001. The genome has an estimated size of about 11.5 Mb, and was found to consist of five replicons with a G+C content of 61.8% (Nazir, Hansen, et al. 2012). The genome of *B. terrae* BS001 is extremely large compared with the genomes of other *Burkholderia* species, that is, *B. rhizoxinica* HKI454 (3.75 Mb) (Lackner, Moebius, Partida-Martinez, et al. 2011), *B. phytofirmans* PsJN (8.21 Mb) (Mitter et al. 2013), and *B. phymatum* STM815 (8.68 Mb). The total number of predicted coding sequences (CDSs) was 12,047, compared with 4,146 in the genome of *B. rhizoxinica* HKI454, 8,043 in that of *B. phytofirmans* PsJN, and 8,434 in that of *B. phymatum* STM815 (table 1). A relatively high number of CDSs, amounting to 38% of the genome, was predicted to encode proteins of unknown function.

**Table 1:** Comparison and General Features of *Burkholderia* Genomes

Bacterial specie	*B. terrae* BS001	*B. rhizoxinica* HKI454	*B. phytofirmans* PsJN	*B. phymatum* STM815
Genome size (Mb)	11.5	3.75	8.21	8.68
GC content (%)	61.8	60.7	62.2	62.3
Number of CDSs	12,047	4,146	8,043	8,434
tRNAs	51	59	63	62
rRNA operons	4	9	18	16

The giant genome size and dense gene coverage suggest that strain BS001 disposes of a suite of lifestyle choices in the soil that are selected as a result of the conditions that may reign locally in the microhabitat it occupies in soil. In previous studies, we found a (one-sided) correlation between fungal-interactivity in soil bacteria and the presence of 1) motility traits and 2) a T3SS. In the following section, we highlight the ecological features of *B. terrae* BS001 in soil and explore the genome for the presence of these as well as other characteristics that may correlate with the (fungal-interactive and otherwise) lifestyle in soil.

### Ecological Features that Presumably Drive the Genome Structure of *B. terrae* BS001

Salient ecological features of *B. terrae* BS001 in soil and the mycosphere are summarized in supplementary table S1, Supplementary Material online. *Burkholderia terrae* was found to be a key inhabitant of the mycosphere of *Laccaria proxima* in the field, whereas *B. terrae* BS001 was an avid migrator with growing *Lyophyllum* sp. strain Karsten mycelium. The rapid migration along the mycelial growth front endowed strain BS001 with the remarkable capacity to successfully reach novel (remote) microhabitats in the soil (Warmink and van Elsas 2009). Moreover, strain BS001 revealed a migration helper effect, as it stimulated the comigration of the nonmigrator *Dyella japonica* BS003 along with the fungal hyphae (Warmink et al. 2011). The organism further showed avid biofilm formation around hyphae of the fungal host. The extracellular polysaccharides constituting the biofilm are thought to act like a shield against antifungal agents (Warmink and van Elsas 2009), allowing protection of the fungal host. *Burkholderia terrae* BS001 was further found to be able to comigrate with a range of other selected soil fungi through soil, allowing the notion that it is a “generalist” migrator. The fungal-interactive capacity was suggested to involve bacterial motility as well as the activity of a T3SS (Warmink and van Elsas 2009). Moreover, considering resource utilization, *B. terrae* BS001—much like *Variovorax paradoxus* HB44—was found to grow on *Lyophyllum* sp*.* strain Karsten released glycerol (tested in liquid microcosms). The organism was even able to trigger the release of glycerol from host cells by an as-yet-unknown mechanism (Boersma et al. 2010; Nazir et al. 2013). Furthermore, it grew avidly on glycerol as the sole carbon source (data not shown). In this sense, *B. terrae* BS001 is likely to be an excellent competitor in soil in competitive situations where carbon sources such as glycerol become available. Finally, *B. terrae* BS001 was indicated to affect the physiology of *Lyophyllum* sp. strain Karsten, including the induction of a secretome that contained high levels of glycerol, next to the inhibition of primordium formation by fungal mycelial mats in the microcosms (Nazir et al. 2013).

### Core and Pan Genome Analyses

**Fig. 1.—:**
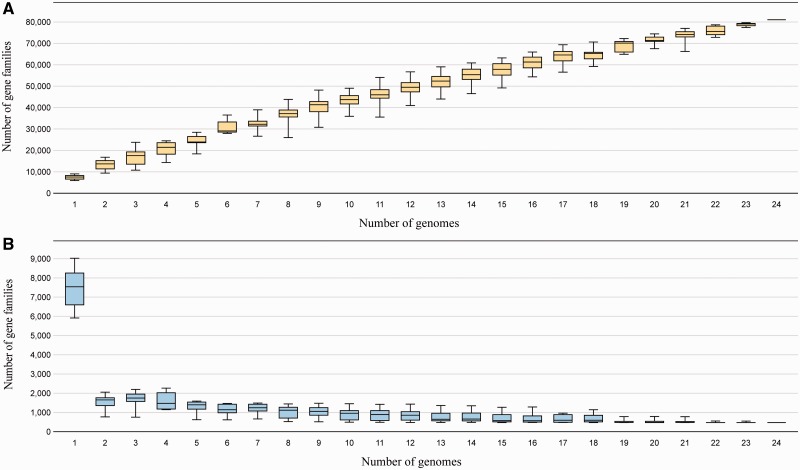
Core–pan genome size evolution. (*A*) Pan genome: Pan genome size is directly proportional to the number of genomes. (*B*) Core genome: Core genome size is inversely proportional to the number of genomes.

We used the “MicroScope” platform (Vallenet et al. 2013) to determine the genetic landscape of the *B. terrae* BS001 genome, taking as references the available (MicroScope) genomes of 23 other *Burkholderia* species, that is, *B. phymatum* STM815, *B. phytofirmans* PsJN, *B. rhizoxinica* HKI454, *B*. *glumae* (strains; AU6208, LMG 2196, BGR1, and 3252-8), *Burkholderia* sp. (strains; CCGE1002, CCGE1003, and TJ149), *B. lata* 383, *B. kururiensis* M130, *B. thailandensis* E264, *B. mallei* ATCC 23344, *B. pseudomallei* K96243, *B. cenocepacia* AU 1054*, B. phenoliruptrix* BR3459a*, B. ambifaria* AMMD, *B. gladioli* (strains; 3848s-5 and BSR3), *B. vietnamiensis* G4, *B. multivorans* ATCC 17616, and *B. xenovorans* LB400. First, *B. terrae* BS001 had the largest genome of all (11.5 Mb compared with 9.7 Mb for *B. xenovorans* LB400), indicating a large accessory gene part. The pan genome across these genomes is defined as the sum of the core genome (genes present in all twenty four organisms), the variable genomes (genes found in some organisms while being absent from the others) and the strain-specific genes (Medini et al. 2005), whereas the core genome is the collective number of shared genes between all genomes. The analysis revealed a total of 180,124 CDSs to be present in the pan genome of the 24 genomes, which were divided over 81,027 MicroScope gene families (MICFAM; see Materials and Methods section). The core genome across all *Burkholderia* species comprised 472 gene families; however, the exact number of core CDSs was different across the compared genomes, as a result of some core CDSs having more than one copies. Thus, strain BS001 was found to have a core of 523 CDSs as several core genes had paralogs. The 523 CDSs core genome of strain BS001 encompassed only 4.34% of its total genome. The remaining 95.66% (11,522 CDSs) thus constituted the variable or accessory part. Of these, there were 6,099 (50.63%) strain-specific CDSs in strain BS001 whereas the remainder (∼5,000) were volatile, meaning that they were present across a limited number of genomes (supplementary table S2, Supplementary Material online). Clearly, as found by others, we also found that the sizes of the core and pan genomes depended strongly on the number of genomes analyzed, resulting in shrinking core and expanding pan genomes with depth of genome sampling (fig. 1). Also, across the *Burkholderia* strains that were analyzed, the pan genome should be considered to be open, as, with every new genome sequenced, several hundreds of novel genes are found (data not shown). Our analysis further revealed that the core genome across all 24 *Burkholderia* genomes consisted of CDSs encoding key cellular functions such as DNA recombination, replication, metabolism, transcription, translation, glycolysis, amino acid activation (tRNAs), chaperoning, RNA modification, transcription regulation, DNA repair, fatty acid biosynthesis, peptidoglycan biosynthesis, posttranslational modification, and cell division. Overall, the level of homology (translated amino acid sequences) of the genome of *B. terrae* strain BS001 with these genomes was between 55% and 60%.

### Phylogeny and Synteny Groups

**Fig. 2.—:**
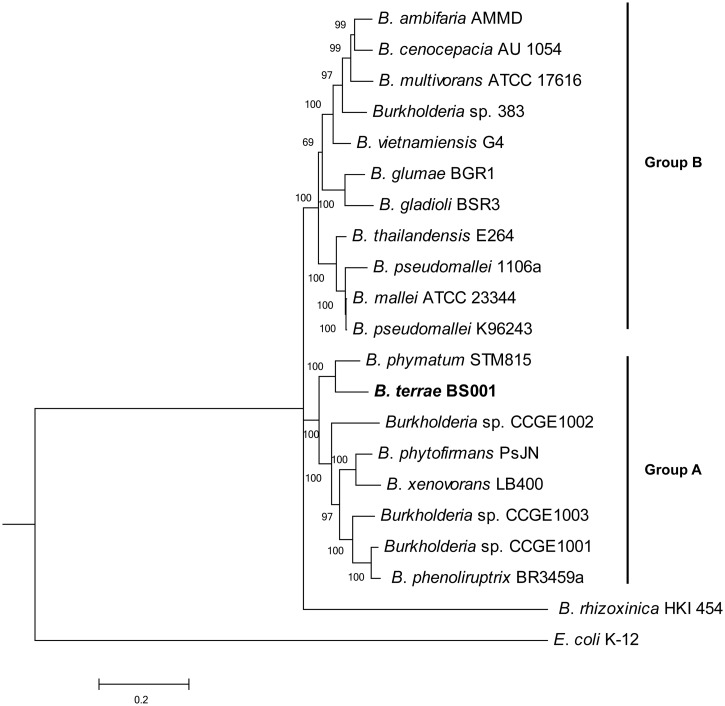
A maximum-likelihood tree illustrating the relationship of selected *Burkholderia* species. The tree is based on alignment of seven concatenated core genes (*aroE, dnaE, groeL, gyrB, mutL, recA,* and *rpoB*) and was generated using MEGA. *Burkholderia terrae* BS001 falls within group A and is closely related to *B. phymatum* STM815. Bootstrap values (more than 50%) are shown at each node. Group A and group B are defined by Estrada-de los Santos et al. 2013.

**Fig. 3.—:**
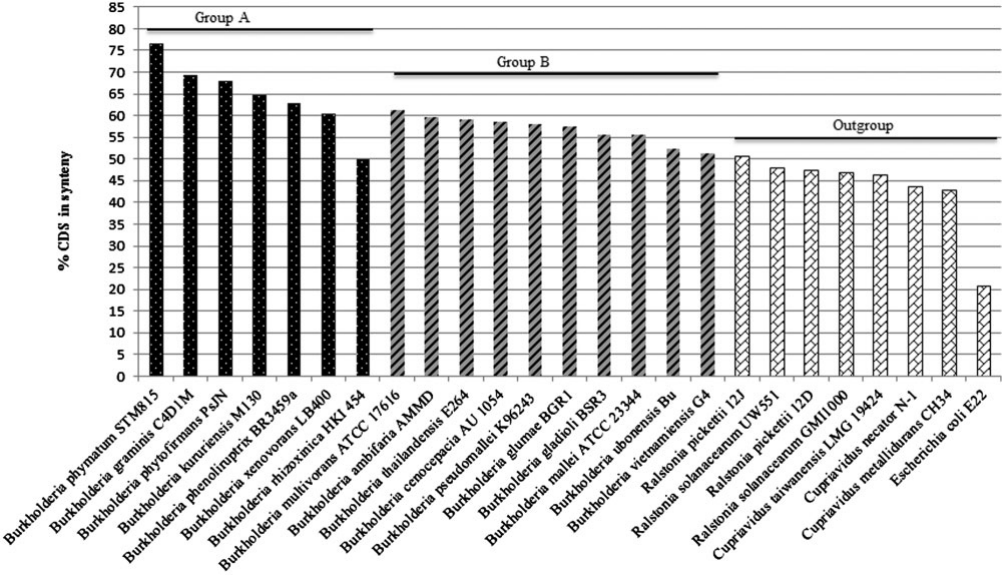
Synteny groups. Syntenous CDSs (%) of *Burkholderia* species from group A and group B, compared with *B. terrae* BS001, are shown. *Ralstonia*, *Cupriavidus*, and *Escherichia coli* species are included as outgroups.

We first constructed a maximum-likelihood tree on the basis of seven concatenated genes for representative *Burkholderia* species from groups A (plant/soil related, nonpathogens) and B (pathogens) (Estrada-de los Santos et al. 2013) to locate the position of strain BS001. Indeed, strain BS001 belonged to group A, being very closely related to *B. phymatum* STM815 (fig. 2). In this analysis, we further found that *B. rhizoxinica* HKI 454 could not be related to any of the other *Burkholderia* groups, as also observed by Estrada-de los Santos et al. (2013). We then performed an analysis of synteny between the strain BS001 genome versus selected available genomes in the NCBI (National Center for Biotechnology Information) RefSeq (reference sequence) database. The analyses (fig. 3) revealed that the *B. phymatum* STM815 genome had highest CDSs synteny to the BS001 genome, that is, 76.78% (fig. 2), followed by *B. graminis* C4D1M, *B. phytofirmans* PsJN, and *B. kururiensis* M130 (69.47%, 67.97%, and 64.79%, respectively). These four *Burkholderia* species are members of *Burkholderia* group A (Estrada-de los Santos et al. 2013) to which *B. terrae* BS001 also belongs (Nazir, Zhang, et al. 2012). A trend of decreasing % CDSs synteny was observed as we moved from *Burkholderia* group A members to those of group B, that is, *B. cenocepacia* AU 1056 (58.66%), *B. pseudomallei* K96243 (58.1%), *B. mallei* ATCC 23344 (55.49%), and *B. vietnamiensis* G4 (51.34%) (fig. 3). The levels of synteny of the outgroups, that is, *Ralstonia, Cupriavidus*, and *Escherichia*, with the genome of the strain BS001, were much lower. These findings suggest that the conservation of synteny was highest among members of the group A *Burkholderia* and it gradually decreased as the phylogenetic distance increased.

### Secretion Systems and Effector Proteins

Secretion systems such as the T3SS may be important for bacteria when these interact with hosts such as fungi (Lackner, Moebius, Hertweck, et al. 2011). We investigated the genome of *B. terrae* BS001 for the presence of (protein) secretion systems, which have a potential role in the interaction with *Lyophyllum* sp. strain Karsten. Basically, we found all known protein secretion systems, that is, the type 1 secretion system (T1SS) through to the type 6 secretion system (T6SS), in the genome of *B. terrae* BS001. The T1SS is normally composed of three constituents, including ATP-binding cassette (ABC) transporters, membrane fusion proteins, and outer membrane proteins (Delepelaire 2004; Holland et al. 2005). The BS001 genome harbored ABC transporters and related proteins that are part of the T1SS (discussed later in detail).

The type 2 secretion system (T2SS) (constituted of 9–11 genes), which is used to translocate a wide range of proteins, was present as three gene clusters, numbered 1 (AKAUv1_257005–AKAUv1_2570061), 2 (AKAUv1_2570149–AKAUv1_2570159), and 3 (AKAUv1_790112–AKAUv1_790123) across the genome. The latter (11-genes) cluster was completely syntenous and highly homologous (>80% amino acid identity [AAI]) to a similar system in *B. phymatum* STM815. The BS001 genome also revealed the presence of two copies of a subtype of the T2SS, the Tad (tight adherence) macromolecular transport system. *Tad* genes encode a machinery that is required for the assembly of adhesive fimbrial low-molecular-weight protein (Flp) pili. The machinery is associated with a so-called widespread colonization island (Tomich et al. 2007). The Flp pili play crucial roles in the formation of biofilms, colonization, and pathogenesis across several bacterial genera (Tomich et al. 2007). The first 14-genes *tad* cluster (AKAUv1_990039–AKAUv1_990052) was highly syntenous and homologous to similar systems in *B. phytofirmans* PsJN (∼60–70% AAI) and *B. phymatum* STM815 (∼70–75% AAI). The second, 13-gene, *tad* copy (AKAUv1_550027–AKAUv1_550039), located at a different position, also had highest synteny with a second system in *B. phymatum* STM815 (>70% AAI) and *B. phytofirmans* PsJN (>55% AAI).

**Fig. 4.—:**
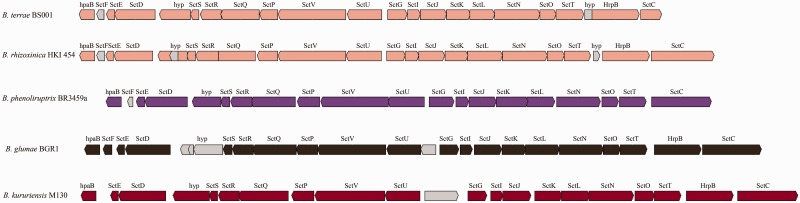
Type 3 secretion system (T3SS). Genetic organization of the T3SS gene cluster found in *Burkholderia terrae* BS001 compared with other *Burkholderia* species including *B. rhizoxinica* HKI454, *B. phenoliruptrix* 3459a, *B. kururiensis* M130 and *B. glumae* BGR1.

**Fig. 5.—:**
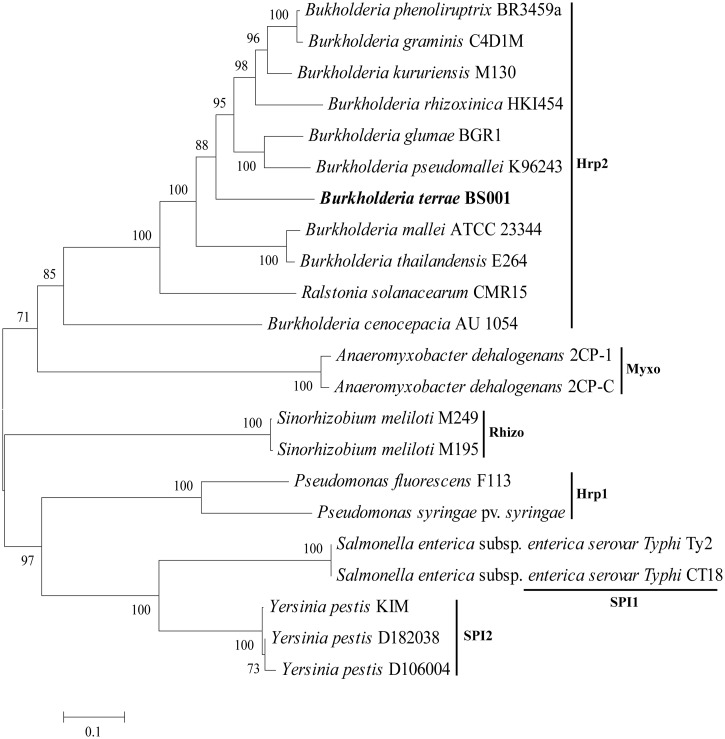
A maximum-likelihood phylogenetic tree. The evolutionary relationship and position of the T3SS of *Burkholderia terrae* BS001 was inferred from concatenated amino acid sequences of eight conserved T3SS genes (*SctS, SctR, SctQ, SctV, SctU, SctJ, SctN*, and *SctT*). The T3SS of *B. terrae* BS001 belongs to the Hrp2 family of T3SS. Hrp2, Myxo, Rhizo, Hrp1, SPI1, and SPI2 are families within T3SSs (Abby and Rocha 2012).

The T3SS has a crucial role in the virulence of several plant and human pathogens (Tseng et al. 2009). It may also be involved in bacterial–fungal interactions, in particular aiding bacteria in their migratory response to extending fungal hyphae in the mycosphere (Warmink and van Elsas 2009). Indeed, the few bacterial types that were able to migrate along with the hyphal front of *Lyophyllum* sp. strain Karsten in soil microcosms were all positive for the presence of the T3SS (Warmink and van Elsas 2009). The T3SS makes membrane-bound complexes that can secrete effector proteins into eukaryotic host cells. The *B. terrae* BS001 genome possesses one T3SS gene cluster, containing 22 canonical T3SS genes (AKAUv1_540178–AKAUv1_540199). Out of these, nine were found to be highly conserved, making up the proposed core apparatus (*SctSRQVUJNTC*), however without a canonical gene for the “needle.” Interestingly, the system was found to be highly syntenous and homologous (60–65% AAI) to the T3SS of the fungal-interactive strain *B. rhizoxinica* HKI454 as well as that of *B. glumae* BGR1 (fig. 4). Remarkably, a CDS with unknown function (AKAUv1_540179) was found, of which the location and length compelled us to argue that it encodes a needle protein. Based on PSIPRED predicted α-helix structures, we assume that this CDS is a functional homolog of *SctF* (*B. rhizoxinica* HKI454) or its *Ralstonia* counterpart *HrpY* (Lackner, Moebius, Hertweck, et al. 2011)*.* To understand the evolutionary position of the T3SS of strain BS001, we constructed a phylogenetic tree (fig. 5) using eight concatenated core genes (*SctS, SctR, SctQ, SctV, SctU, SctJ, SctN,* and *SctT*) that were conserved across the tested genomes. The analysis supports the conclusion that the T3SS of *B. terrae* BS001 belongs to the so-called Hrp2 family, to which most of the *Burkholderia* and *Ralstonia* T3SSs belong (Abby and Rocha 2012). The T3SSs of *B. terrae* BS001 and *B. rhizoxinica* HKI454 might fulfill similar roles. Both strains interact actively with fungal hosts and there is evidence that T3SSs play a role in the interaction in both cases (Lackner, Moebius, Hertweck, et al. 2011;  Warmink and van Elsas 2008).

**Fig. 6.—:**
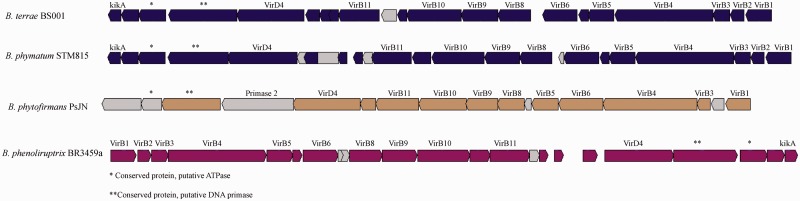
Type 4 secretion system (T4SS). The gene organization of T4SS-I (gene cluster 1) of *Burkholderia terrae* BS001 compared with the T4SSs *B. phymatum* STM815, *B. phenoliruptrix* 3459a, and *B. phytofirmans* PsJN is shown.

The type 4 secretion system (T4SS) is responsible for secreting proteins and, most importantly, nucleic acids (coupled to proteins; nucleoproteins) across the inner and outer membranes into recipient cells or into the external milieu. It plays a key role in the transfer of plasmids or integrated chromosomal elements, next to being involved in virulence on animals (protein secretion, e.g., in *Helicobacter pylori*) or plants (plasmid transfer, e.g., in *Agrobacterium tumefaciens*) (Dale and Moran 2006). Remarkably, the genome of *B. terrae* BS001 was found to contain three T4SS gene clusters. The first one, T4SS-I (20 CDSs; AKAUv1_3130024–AKAUv1_3130044), with all canonical T4SS functions (fig. 6), is present on the 70.42-kb region of genome plasticity (RGP) RGP76. The predicted *virB10* gene of T4SS-I had very high AAIs with similar genes from a *Burkholderia* sp. BT03 plasmid (94%) and the *B. phymatum* STM815 genome (71%). Furthermore, non-T4SS open reading frames (ORFs) on RGP76 were predicted to encode a DNA circulation family protein, DNA replication protein *tnpB*, transposase *tnpA*, IS5 transposase *insH*, next to seven integrases, including two phage family integrases. Further, at the downstream boundary of RGP76, four transposases and two integrases were localized. The T4SS was completely syntenous and highly homologous (70–75%) with a *B. phymatum* STM815 T4SS located on plasmid pBPHY02 (595,108 bp). Moreover, the regions flanking RGP76 also contained CDSs potentially involved in gene mobility, that is, ORFs similar to the typical plasmid replicon genes *parA, parB, parD, parE, mobB, mobC, repB, korC*, and *klcA.* RGP76 further carried several accessory genes, that is, those predicted to function as a putative xanthine dehydrogenase/carbon monoxide dehydrogenase acceptor (AKAUv1_3070015, AKAUv1_3070016) and molybdenum binding xanthine dehydrogenase (AKAUv1_3130017). The remaining accessory ORFs were predicted to encode conserved proteins of unknown functions.

A second system, T4SS-II, containing 13 genes, was found to be present elsewhere in the genome (AKAUv1_1810006–AKAUv1_1810019). This cluster had a gene composition different from the first one, as it contained the *virB8, virB9,* and *virB10* genes but lacked other *vir* genes. Instead, it contained CDSs for lytic transglycosylase (two CDSs), *pilL*, toxin coregulated pilus biosynthesis protein Q, *pulD, pulE, pulF* (T2SS), and conserved hypothetical proteins genes.

The third system, T4SS-III, (AKAUv1_1120025–AKAUv1_1120032) contained only eight T4SS related genes. T4SS-III contained *virB4, traG, traL* (relaxase) *virB6, trbJ, trbD, trbC,* and *trbB* genes. However, T4SS-III lacked the canonical *virB10* gene. In order to assess the origin of the former two T4SSs, we performed a phylogenetic analysis of their respective *virB10* gene amino acid sequences. The analysis showed that T4SS-I is highly similar (72% AAI) to T4SSs of both *B. phymatum* STM815 and *B. phenoliruptrix* BR3459a. The T4SS-II, being very different from T4SS-I (22% homology), was closely related (76% AAI) to a T4SS of *Burkholderia* sp. SJ98 (supplementary fig. S1, Supplementary Material online).

The *B. terrae* BS001 genome further revealed the presence of four copies of predicted T6SSs, numbered T6SS-I through T6SS-IV (12–25 genes). T6SS-1 (16 genes; AKAUv1_650058–AKAUv1_650074) had highest synteny and homology (>40%) with a genomic region of *B. gladioli* BSR3. T6SS-II (19 genes; AKAUv1_1080195–AKAUv1_1080214) was highly syntenous and homologous (85–95%) with a similar cluster of *B. phymatum* STM815. T6SS-III and T6SS-IV comprised lower numbers of genes, that is, 8 (AKAUv1_1680089–AKAUv1_1680097) and 5 (AKAUv1_920001–AKAUv1_920005), respectively. T6SS-III had highest synteny with clusters in *B. phenoliruptrix* BR3459a and *B. kururiensis* M130 (both 55–60% homology). T6SS-IV, found on RGP25, was syntenous with five-genes clusters (40–50% homology) in *B. glumae* strains BGR1 and AU6208 and *Burkholderia* sp. CCGE 1002 (supplementary fig. S2, Supplementary Material online).

The *B. terrae* BS001 genome also contained several genes for signal peptide bearing proteins (supplementary table S3, Supplementary Material online) that may be exported in a *sec*-dependent fashion. Among these, the so-called type II secreted proteins fall in the category of lytic and toxin-related proteins (Cianciotto 2005). Furthermore, putative *ppiA* (encoding peptidyl–prolyl *cis*–*trans* isomerase A; rotamase A; AKAUv1_720068), next to genes for parvulin-type peptidyl–prolyl *cis*–*trans* isomerase (AKAUv1_1270066), and peptidyl–prolyl *cis*–*trans* isomerase (AKAUv1_1390107) were found. The latter protein may have pathogenicity-enhancing abilities in *Legionella pneumophila* and *Xanthomonas campestris* pv. *campestris* 8004 (Fischer et al. 1992; Zang et al. 2007). It has also been reported to exist in *B. rhizoxinica* HKI454 (Lackner, Moebius, Partida-Martinez, et al. 2011). We also found gene *iagB* (AKAUv1_790111) and its three duplicates (45–65% AAI), which produces an invasion protein and may be involved in the invasion of eukaryotic host cells by *Shigella flexneri* and *Salmonella enterica* serovar typhi (Allaoui et al. 1993; Miras et al. 1995). Three genes predicted to encode ankyrin (AKAUv1_540128, AKAUv1_920061, AKAUv1_1270092) and tetratricopeptide (TPR) repeat containing proteins were also present in the BS001 genome. These proteins may be involved in protein–protein interactions as host interactive factors (Edqvist et al. 2006). Both ankyrin and TPR repeat proteins have also been reported in *B. rhizoxinica* HKI454 (Lackner, Moebius, Partida-Martinez, et al. 2011).

Putative T3SS secreted effector proteins were then searched for using www.effectors.org (last accessed June 26, 2014) (Jehl et al. 2011) (supplementary table S4, Supplementary Material online), which were analyzed with respect to the putative interaction of *B. terrae* BS001 with *Lyophyllum* sp. strain Karsten. These effectors need confirmation, as we ignore their functions at this moment. Nevertheless, we found putative phytoene synthase (AKAUv1_920106), of the squalene–hopene cyclase enzyme family and may be involved in the biosynthesis of terpenoids. This is consistent with the presumption that terpenoids mediate the interactions of *B. terrae* BS001 with its host, like reported for *B. rhizoxinica* HKI454 in its interaction with the fungal host (Lackner, Moebius, Partida-Martinez, et al. 2011). Among the predicted effectors we also found flagellin proteins (coined pathogen-associated molecular patterns) both in plants and in mammals (Ronald and Beutler 2010; Wei et al. 2013).

### Pilus, Biofilm Formation, Motility, and Insecticidal Toxin Complexes

The genome of *B. terrae* BS001 further carries gene clusters predicted to encode flagellar biosynthesis, biofilm formation, motility, and (seven genes) type 4 pili. The latter pili may play a role at fungal surfaces, as, once attached to the surface, bacteria can “walk” on it by employing the action of such pili (Conrad 2012). Genes that encode such pili, that is, *pilMNQ* (AKAUv1_1170009, AKAUv1_1170010, AKAUv1_1170012), were present in a cluster. We also found pilin precursor gene *PilA* (AKAUv1_2170003), signal peptidase *PilD* (AKAUv1_2940033), NTP-binding protein *PilB* (AKAUv1_2940037), and *PilC* (AKAUv1_2940035) in the genome.

Bacterial biofilm formation is dependent on the ability to produce extracellular matrix which may be composed of polymers like poly-beta-1,6-*N*-acetyl-d-glucosamine (PGA) (Izano et al. 2007; Bosse et al. 2010). We found several ORFs predicted to be involved in PGA production, that is, *pgaA* (PGA synthesis protein; AKAUv1_1000016), *pgaB* (PGA synthesis lipoprotein; AKAUv1_1000017), and *pgaC* (PGA synthesis *N*-glycosyltransferase; AKAUv1_1000018). In addition, two other biofilm synthesis gene clusters (both representing a Pel operon homolog) were identified. The Pel operon provides a scaffold in the early stage of biofilm formation by *Pseudomonas aeruginosa* PA14, (Colvin et al. 2011). Pel is thought to encompass a glucose-rich polysaccharide polymer that is encoded by a seven-genes operon (*pelA–F*) (Friedman and Kolter 2004). Cluster 1 comprised *pelABCDEFGA* (AKAUv1_2280066–AKAUv1_2280073), and cluster 2 *pelGFEDCBA* (AKAUv1_100003–AKAUv1_100009). Thus the Pel region may have undergone a recent duplication followed by some reshuffling, as evident from our analysis. Two genes, taken as proxies in the two systems, that is, *pelG* and *pelF,* were 61.84% and 64.69% identical, respectively, whereas *pelABCDE* had lower (43.52%, 36.33%, 47.9%, 40.08%, and 45.55%) AAI. An extensive screening further revealed the presence of ten other CDSs (i.e., 4 *epsA*, 4 *epsB,* and 2 *epsP*) in the genome that are potentially involved in the biosynthesis of other exopolysaccharide (EPS). Overall, the presence of the *pel* gene clusters reflects another capacity of *B. terrae* BS001 to form biofilms during its interaction with *Lyophyllum* sp. strain Karsten (as well as other hosts) in soil.

Moreover, the *B. terrae* BS001 genome harbors one gene cluster that encodes flagellar biogenesis proteins. The cluster revealed the presence of 46 genes (AKAUv1_120002–AKAUv1_120048) in a region that was highly syntenous with regions of the STM815 (80–90% AAI) and PsJN (75–85% AAI) genomes. Moreover, the BS001 genome contained a gene cluster comprised 11 genes (AKAUv1_790001–AKAUv1_790011) associated with bacterial chemotaxis and motility. Again, this cluster was syntenous with a similar one in strain STM815 (85–95% AAI). Elsewhere in the genome, another nine flagellar genes (*flhA, flhB,* 2 [*flhC*]*,* 3 [*flhD*]*, fliC*, and *fliD*) were found, next to a seven-genes chemotaxis/motility cluster (AKAUv1_920023–AKAUv1_920030).

Interestingly, the genome of *B. terrae* BS001 harbored two ORFs (AKAUv1_2130030 and AKAUv1_2130031) coding for a putative insecticidal toxin complex, as also reported to be present on the 822-kb megaplasmid pBRH01 in *B. rhizoxinica* HKI454 (Lackner, Moebius, Partida-Martinez, et al. 2011). We analyzed the putative horizontal mobility of such ORFs. Indeed, the two ORFs formed part of the largest genomic island, RGP53 (102,873 bp), suggesting that they may have been acquired horizontally. Thus strain BS001 might have insect pathogenicity traits, indicating its putative interaction with soil insects.

### Genomic Islands across the Genome of *B. terrae* BS001

We employed “MicroScope” (Vallenet et al. 2013) to predict the presence of RGPs in the BS001 genome. The analysis predicted the existence of 79 RGPs, which were scattered across the genome (supplementary table S5, Supplementary Material online). The total size of the collective RGPs was 1,896,071 bp, accounting for 16.48% of the genome. Among the RGPs, we found the aforementioned 70.42 kb plasmid-like RGP76 (AKAUv1_3070018–AKAUv1_3130074). An overview of ten important RGPs with plasmid-related and functional genes is given in supplementary table S6, Supplementary Material online.

Briefly, on RGP34, we identified two ORFs (AKAUv1_1280007 and AKAUv1_1280008) encoding putative addiction proteins (toxin and antitoxin). In this region, four ORFs (AKAUv1_1930029, AKAUv1_1930030, AKAUv1_1230100, and AKAUv1_1230101) encoding *HigA, HigB* (plasmid maintenance protein), *StbD* and *StbE* (stabilization proteins) were found just outside of the RGP. Similarly, putative plasmid segregation antitoxin *CcdA* and *CcdB* toxin proteins are encoded by ORFs AKAUv1_2410015 and AKAUv1_2410016, both located outside plasticity regions. On the other hand RGP61 harbored two ORFs, AKAUv1_2490012 and AKAUv1_2490013, which encoded *CcdB* and *CcdA*. Downstream of these genes, the region harbored an ORF (AKAUv1_2490015) predicted to encode a protein having 39% (coverage 77%) homology with cryptic plasmid protein A from *Neisseria gonorrhoeae* NCCP11945. Interestingly, it also revealed an ORF (AKAUv1_2490048) that encoded a beta-lactamase (with 55% and 51% similarity to a similar protein in *Bradyrhizobium* SG-6C and *R. etli* CFN 42, respectively). Furthermore, we identified putative *parE* and *parD* genes (AKAUv1_2860003 and AKAUv1_2860004) in RGP71, whereas another ORF (AKAUv1_330002) encoding “prevent host death” family protein; *Phd* was present outside the RGPs. We also identified *HigB* and *HigA* (AKAUv1_360025 and AKAUv1_360026) as well as *MazE* and *MazF* addiction modules (AKAUv1_920172 and AKAUv1_920173), outside the predicted RGPs. Such toxin–antitoxin system proteins play a role in mediating stability of plasmids by imposition of an addiction mechanism to the host (Arcus et al. 2005). They may also play a role in mediating growth arrest during stress situations (Gerdes et al. 2005).

The largest RGP, RGP53, was found to span 102,873 bp (AKAUv1_2120017–AKAUv1_2140005), carrying ORFs for seven transposases and one integrase. A screen for functional genes in this RGP revealed the presence of one gene *sip* encoding a siderophore interacting protein (SIP) (AKAUv1_2130016) highly similar to a *sip* from *Burkholderia* sp. BT03 (99%). The gene had a duplicate downstream (AKAUv1_2130093), having (99%) similarity with *sip* of *Burkholderia* sp. BT03. Another ORF (AKAUv1_2130052) was predicted to encode invasion plasmid antigen J (*IpaJ*); it had 35% homology with *IpaJ* from *S. flexneri* 2002017. RGP53 also carried a gene for type III effector protein *HopJ* (AKAUv1_2130053); it was 53% homologous to similar gene from *Salinivibrio costicola*. It appears to furnish a wealth of secondary functions to its host, which may be useful in a wide range of ecological conditions. The clear mobility and divergent accessory nature of RGP53 might suggest that it is an ecological flexibility island.

### Primary Metabolites and Metabolic Pathways

We used the “MicroCyc” metabolic database (Caspi et al. 2010) to compare the metabolic potential in the genome of *B. terrae* BS001 with that of other *Burkholderia* genomes (supplementary table S7, Supplementary Material online).

Based on the values computed for pathway completion, hierarchical cluster analysis was performed for the *B. terrae* BS001 genome next to 23 other genomes including next-of-kin *Burkholderia* species (supplementary fig. S3, Supplementary Material online). The analysis revealed that the *Burkholderia* strains tended to group together, *B. terrae* BS001 being close to *B. kururiensis* M130 and *B. phymatum* STM815, whereas *B. phytofirmans* PsJN grouped with *B. vietnamiensis* G4 and *B. ambifaria* AMMD. Interestingly, *B. rhizoxinica* HKI454 grouped outside of the main *Burkholderia* cluster; however, this might have been compromised by its smaller genome size.

*Burkholderia terrae* BS001 clearly invested in diverse primary metabolisms, as evidenced from the presence of more than 1,200 putative CDSs for carbohydrate metabolism (Nazir, Hansen, et al. 2012). Specifically, we found a putative glycerol kinase, *glpK* (AKAUv1_1300029), which was preceded by a glycerol-3-phosphate dehydrogenase (*glpD,* AKAUv1_1300028).

**Fig. 7.—:**
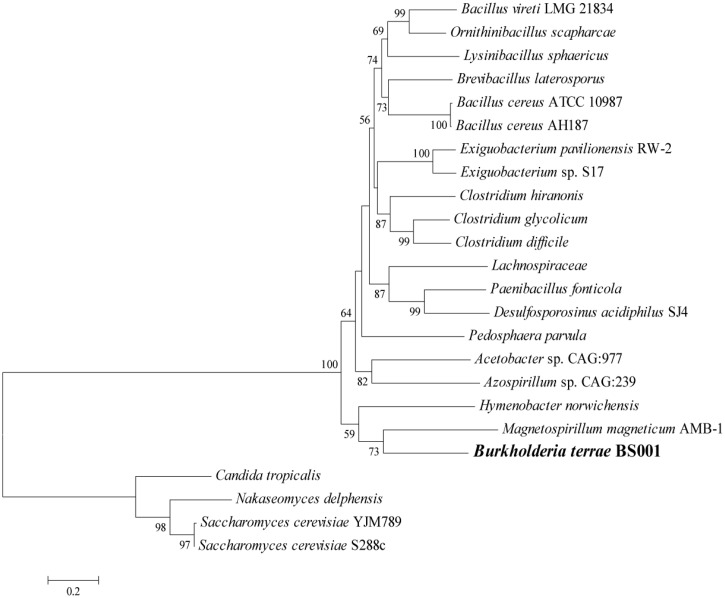
A neighbor joining tree of glycerol uptake gene (*GUP*) of *Burkholderia terrae* BS001 and close relatives. Based on the amino acid sequence of *GUP* gene, *B. terrae* BS001 *GUP* clustered with *a similar gene product of Magnetospirillum magneticum* AMB-1. *GUP* amino acid sequences of *Saccharomyces cerevisiae* strains s288c, YJM 789, *Candida tropicalis* and *Nakaseomyces delphensis* are taken as an outgroup. Bootstrap values (more than 50%) are shown at the nodes.

Remarkably, in the *B. terrae* BS001 genome, we found a gene for a specific glycerol uptake family transporter, denoted GUP. This system was absent from all other *Burkholderia* genomes and it is actually typical for the yeast *Saccharomyces cerevisiae.* In this organism, active uptake of glycerol occurs mainly by *GUP* 1 and its close homolog *GUP* 2, which both encode multimembrane-spanning proteins (Holst et al. 2000). Interestingly, the gene for *GUP* (AKAUv1_1930108) was present on RGP47, showing highest AAI (49%) with *Magnetospirillum magneticum* AMB-1 (fig. 7). The upstream ORF AKAUv1_1930107 encodes protein of unknown function that also shares 25% AAI with a hypothetical protein from *M. magneticum* AMB-1. The unique presence of this *GUP* gene in *B. terrae* BS001 is consistent with the hypothesis that fungal-exuded glycerol (Nazir et al. 2013), which feeds the bacterium, may be taken up through it. The presence of this system on RGP47 may indicate that the bacterium acquired and fixed it in its quest to thrive in the mycosphere at the expense of exuded glycerol.

Genes for glycerol degradation pathways I and V were predicted to be present in the genome of *B. terrae* BS001. In comparison with the comparator strains, glycerol degradation pathway V was only complete in *B. terrae* BS001, whereas it was incomplete in strains PsJN and STM815 while strain HKI454 completely lacked it. Pathway V encompasses two reactions, and only the *B. terrae* BS001 genome carried ORFs (AKAUv1_760319, AKAUv1_2440011, and AKAUv1_1890037) encoding enzymes catalyzing these. The first two ORFs are duplicates of each other, having 42.9% sequence identity.

Strikingly, the BS001 genome further carried ORF AKAUv1_1970009, which is predicted to encode a tyrosinase (involved in l-dopachrome biosynthesis), present on RGP49. We analyzed the potential phylogenetic history of this gene using a maximum-likelihood tree (supplementary fig. S4, Supplementary Material online) with closest hits in the database. Blasting the whole NCBI database only yielded four close hits, with highest AAI (73%) with *Anaeromyxobacter dehalogenans* 2CP-1. The ORF directly upstream in BS001, AKAUv1_1970008, was predicted to encode a twin-arginine signal translocation protein (Tat). This gene also occurred (55% AAI) directly upstream in *A. dehalogenans* 2CP-1. However, the region downstream of ORF AKAUv1_1970010 was different. The presence of the *Tat* gene upstream of the tyrosinase gene is consistent with the fact that the tyrosinase may require a signal peptide containing transactivator for its export and translocation (Berks et al. 2000; Schaerlaekens et al. 2001). This commonality between the rather unique *B. terrae* system and one in *A. dehalogenans* is striking. The latter organism belongs to a very different bacterial clade (delta-Proteobacteria) than *B. terrae* BS001; however, it occurs in the same habitat (soil) and is a very motile (gliding) bacterium (Thomas et al. 2008). Moreover, its mosaic genome has been postulated to be composed of parts coming from diverse proteobacteria. Based on this analysis and the uniqueness of the commonality, we postulate that the gene for tyrosinase may have been acquired from an organism like *A. dehalogenans* 2CP-1 through horizontal gene transfer. l-Dopachrome is the building block of melanin, which can protect against ultraviolet radiation (Liu et al. 2004). Moreover, melanin in the cell wall of *Aspergillus nidulans* has been correlated to resistance against digestion by chitinases, glucanases, and proteases (Kuo and Alexander 1967). Bacterial melanin has been shown to interact with double-stranded DNA and its cellular localization may inhibit cell metabolism (Geng et al. 2010). Bacterial tyrosinases may also act on particular phenolic compounds, such as chlorophenols (Marino et al. 2011) and fluorophenols (Battaini et al. 2002). The role of the unique tyrosinase found in the *B. terrae* BS001 genome is not well understood, but it may serve the organism to cope with stressful environments and detoxify fungal/plant phenolic compounds.

Furthermore, the *B. terrae* BS001 genome revealed an enormous biosynthetic potential for a wide range of amino acids, see supplementary table S6, Supplementary Material online, indicating its flexibility in synthesizing the essential cellular building blocks of different kinds.

### Plant-Interactive Traits

In the *B. terrae* BS001 genome, we further found biosynthetic potential for plant hormones, that is, genes for ethylene biosynthesis pathway III (two out of three enzymes are present) and indole acetic acid (IAA) biosynthesis IV and V, whereas IAA biosynthesis pathway VI is partially present. This may suggest that *B. terrae* BS001 spends (part of) its life occupying a niche close to plants like the rhizosphere. The presence of three copies of a 1-aminocyclopropane-1-carboxylate deaminase (ACC deaminase—*acdS*) gene (AKAUv1_150009, AKAUv1_510080, and AKAUv1_780047), denoted *acdS*-I, *acdS*-II and *acdS*-III, gives support to this hypothesis. Maximum-likelihood phylogenetic analyses showed that *acdS*-III clustered separately from the other two genes and might have been acquired through horizontal gene transfer (HGT). In contrast, *acdS*-I and *acdS*-II may be the result of a duplication very early in evolution (supplementary fig. S5, Supplementary Material online).

Similarly, RGP53 was found to carry two CDSs, here denoted as p*nodW* (putative *nodW* genes) (AKAUv1_2130064 and AKAUv1_2130080) that may act as two-component response regulators, with 81% AAI to a putative two-component response regulator from *P. resinovorans* NBRC 106553. Further analyses revealed that up to 18 paralogs of p*nodW* genes were present across the genome, with similarities of 30–69%. Downstream one ORF (AKAUv1_2130067), we also found a PAS sensor protein (sensor kinase; *nodV*). This sensor kinase had seven paralogs in the genome with AAI of 35–46%. Both *nodV* and *nodW* belong to the two-component regulator family that activates the expression of other nodulation genes at the plant surface and in response to isoflavonoides (Sanjuan et al. 1992; Loh et al. 1997, 2002). We could not find other nodulation genes (such as *nodABCD*); however, elsewhere in the genome we found CDSs similar to *nodN, nodI, nodJ,* and *nodT*. The exact role of the two-component regulators (*nodV* and *nodW*) in strain BS001 is not known yet, but the presence of such an array of *nod* genes allows the hypothesis that *B. terrae* BS001 might also display a plant-interactive lifestyle. On the other hand nitrogen fixation genes were not present in the genome, excluding a classical nitrogen fixation interaction.

### Detoxification Potential

The *B. terrae* BS001 genome revealed great potential to detoxify arsenate, phenylmercuric acetate, methylglyoxal, cyanate, fluoroacetate, and superoxide radicals. The gene encoding arsenate reductase (*arsC*) (AKAUv1_1500002) is present on RGP38. Interestingly, two genes (AKAUv1_1500022 and AKAUv1_1500025) involved in cyanate degradation are located downstream on RGP38. Moreover, ORF AKAUv1_3230012, encoding aliphatic nitrilase that catalyzes a reaction in acrylonitrile degradation, was found to be harbored by RGP78. Another putative ORF, AKAUv1_2920114, was harbored by RGP73. It encodes a putative 2-nitropropane dioxygenase that is involved in alkylnitronate degradation. We also found that strain BS001 is capable of degrading salicylate, anthranilate, benzoate, and catechol. Similarly, proteins for degradation of compounds that are released during the degradation of plant lignin in soil, such as protocatechuate, ferulate and vanillate, as described for *Ralstonia solanacearum* (Genin and Boucher 2004), are represented by ORFs in the genome of *B. terrae* BS001.

The afore analyses indicate that *B. terrae* BS001 has invested considerable genetic luggage in detoxification systems, which may relate to its persistence in ecological niches that are affluent in such compounds and may explain its versatile ecological behavior.

### Membrane Transporter Landscape

**Fig. 8.—:**
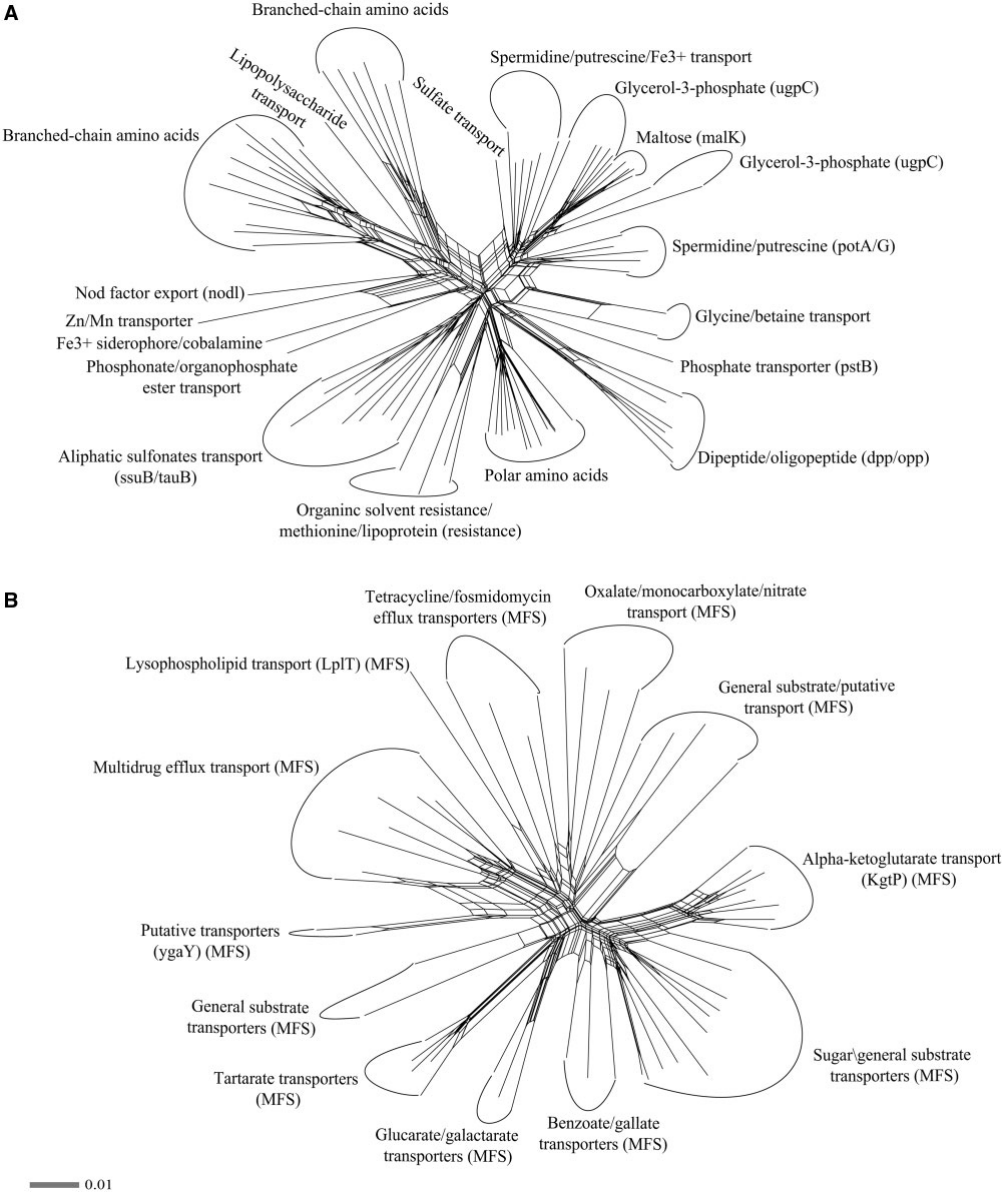
Neighbor-net splits graphs depicting the membrane transporters landscape of *B. terrae* BS001. (*A*) NNet splits graph inferred from alignment of 81 ATP binding component CDSs (amino acid sequences) of ABC (ATP binding cassette) transporters. (*B*) NNet splits graph derived from alignment of 55 MFS (major facilitator superfamily) CDSs (amino acid sequences). Alignments were manually curated and the resulting “nexus” files were analyzed using ProteinMLdist and WAG model (Gamma 4.0) within SplitsTree4 software. The splits indicate that membrane transporters (both ABC and MFS) split out and cluster by their respective functions and that gene duplications are at the basis of membrane transporters diversity. The list of CDS analyzed is given in the supplementary table S10, Supplementary Material online.

As gatekeepers to the outside, membrane transporters are important for metabolic activities. Lifestyle is thought to be a key driver of the evolution in numbers and types of transporters (Gelfand and Rodionov 2007). Transporters can be classified into three different classes based on their structure and mechanism of action, that is, energy-dependent (ATP dependent) membrane pumps, ion channels, and secondary transporters (Nagata et al. 2008). We used TransAPP (Transporter Automatic Annotation Pipeline) in the search for membrane transporters. A total of 1,338 transporter-like ORFs were found (supplementary table S8, Supplementary Material online), and so the membrane transporter landscape of *B. terrae* BS001 was different from that of the other *Burkholderia* strains. *Burkholderia phymatum* STM815 had 935 transporters, *B. rhizoxinica* HKI454 267 while according to Mitter et al. (2013), *B. phytofirmans* PsJN has 1,196 membrane transporters. As the transporter database lists 997 transporters for *B. phytofirmans* PsJN, we used this figure for our analysis. Across these *Burkholderia* species, a relatively constant percentage of the total genomic space is thus dedicated to transporters, and so the larger genomes such as BS001 (11.1%), STM815 (10.8%), and PsJN (12.1%) have more transporters compared with smaller ones such as HKI454 (7.1%). Overall, the ATP-dependent systems represented the bulk of transporters (648), followed by major facilitator family (MFS) (237) membrane transporters. A detailed account of the transporters across all four *Burkholderia* genomes analyzed is given in supplementary table S9, Supplementary Material online. A total of 81 ATP binding component CDSs of ABC transporters as well as 55 MFS CDSs (supplementary table S10, Supplementary Material online) were randomly picked and analyzed using SplitsTree4 (Huson and Bryant 2006). Thus, for both classes of transporters, a neighbor-net splits network was generated in order to depict different splits indicating potential evolutionary events. The ATP binding components of the ABC transporters revealed varying splits and radiations, indicating duplications to serve different functions in the genome (fig. 8*A*). Transporters of branched-chain amino acids clustered together, as opposed to those involved in glycerol-3-phosphate and sugars. Similarly, another split indicated that phosphate, Zn/Mn, Fe^3+^, and nod factor transporters clustered together and are duplicates. Thus, a range of duplication events followed by mutational drift and selection is at the basis of the current diversity of membrane transporters in strain BS001. Similarly, MFS transporters having similar roles clustered together, indicating another suite of duplication events (fig. 8*B*). Thus, expansion of the genome of *B. terrae* BS001 may have been driven mainly by nutritional needs in the light of locally fluctuating nutrient availability, for which a diversity of transporter systems is essential. Ren and Paulsen (2005) indicated that gene duplications might be at the basis of expansion, diversification, and selection of distinct transporters families.

### Secondary Metabolite Biosynthetic Potential

**Fig. 9.—:**
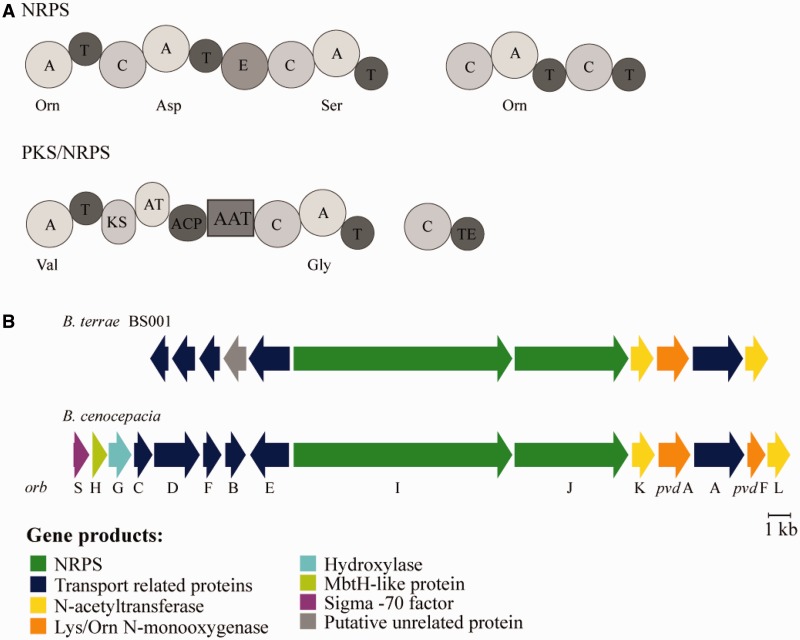
Modular organization of the NRPS (nonribosomal peptide synthetase) and the PKS (polyketide synthase)/NRPS enzymes encoded in the genome of *Burkholderia terrae* BS001. (*A*) Predicted substrates that get activated by the A-domains, are shown. Orn (Ornithine), Asp (Aspartic acid), Ser (Serine), Val (Valine) and Gly (Glycine). A, adenylation domain; T, thiolation domain; C, condensation domain; E, epimerization domain; KS, ketosynthase domain; AT, acetyl transferase domain; ACP, acyl carrier protein; AAT, aminotransferase; TE, thioesterase. (*B*) Comparison of the ornibactin (*orb*) biosynthesis gene cluster of *B. cenocepacia* and the NRPS gene cluster of *B. terrae* BS001. *orb* (S, H, G, C, D, F, B, E, I, J, K, A, and L) and *pvd* (pyoverdine) A and F, are shown.

Many *Burkholderia* species exhibit a high potential for secondary metabolite production (Kang et al. 1998; Partida-Martinez and Hertweck 2005; Partida-Martinez et al. 2007; Knappe et al. 2008; Schmidt et al. 2009; Rohm et al. 2010; Seyedsayamdost et al. 2010; Tawfik et al. 2010; Mahenthiralingam et al. 2011; Franke et al. 2012). As *B. terrae* BS001 possesses an exceptionally large genome, one might predict the ample presence of novel biosynthesis gene clusters. Analyses with antiSMASH (Medema et al. 2011; Blin et al. 2013) revealed the existence of a locus (14,592 bp) that likely encodes a nonribosomal peptide synthetase (NRPS) (AKAUv1_1360004–AKAUv1_1360005) and another one (12,975 bp) coding for a hybrid polyketide synthase (PKS)/NRPS enzyme (AKAUv1_1710055, AKAUv1_1710056, and AKAUv1_1710057) (fig. 8*A*). The NRPS locus (AKAUv1_1360004–AKAUv1_1360005) displays similarity (63% and 60%, respectively) to an NRPS locus of *B. cenocepacia* that is known to encode the siderophore ornibactin (Agnoli et al. 2006). As a mycosphere-inhabiting bacterium, *B. terrae* BS001 is postulated to depend on iron uptake systems under conditions of low iron. However, in the predicted ornibactin-like NRPS locus, four genes (*pvdF* [*N^5^*-hydroxyornithine transformylase], *orbS* [sigma factor], *orbH* [unknown function], *orbG* [α-ketoglutarate-dependent hydroxylases]) were missing. Moreover, several transporter genes (*orbB*, *orbC*, *orbD*, and *orbF*) involved in transport and utilization, differed from those of the ornibactin locus of *B. cenocepacia* (fig. 9*B*).

The modular organization of another cluster—a hybrid PKS/NRPS—is shown in figure 9*A*. Homologs of such hybrid PKS/NRPS gene clusters have been found in other bacterial genomes by BLASTP analyses (Altschul et al. 1990), notably in *Burkholderia* sp. BT03 and *B. phymatum* STM815. However, no secondary metabolite has been described for such gene clusters yet. It would mean a great step forward if the products synthesized by these two clusters could be identified.

The BLAST search for homologs of enzymes of the ribosomally synthesized and posttranslationally modified peptides (RiPPs) pathway revealed no hits, suggesting that *B. terrae* BS001 is not able to produce any such RiPPs.

## Conclusion

We analyzed the genome of the fungal-interactive *B. terrae* BS001, which was recently sequenced (Nazir, Hansen, et al. 2012). The genome is the largest among all hitherto available *Burkholderia* genomes, and it is equipped with a repertoire of genetic systems that encode predicted fungal- as well as plant-interactive traits. We thus identified systems potentially involved in physical interactions (biofilm formation and twitching motility) with fungi, next to protein (as well as DNA) secretion systems (in particular T3SS) that may modulate fungal host physiology and unique genes for glycerol uptake and metabolism. The genome further contains extensive RGPs (16.48% of the genome space), which carry functional genes as well as plasmid-related traits, indicating the presumed great role of horizontal gene transfer in shaping the genome. Moreover, *B. terrae* BS001 apparently invested considerable energy in primary (carbohydrate and amino acid) metabolism, membrane transporters, and even insect-interactive traits, indicating a versatile life style in soil. Such life style may have included (fungal/plant) host-interactive phases interspersed with free-living phases. Concerning the latter, the BS001 genome also contained genes that potentially encode secondary metabolite biosynthesis, which is useful for highly competitive situations that may occur in the soil upon exploration for colonization space. Overall, our analysis unveiled a plethora of ecological features in the genome that provide a firm basis for further studies based on transcriptomics of ecological situations, including interactions with (fungal) hosts.

## Materials and Methods

### Genome Analysis and Annotation

The genome of *B. terrae* BS001 was submitted to MicroScope platform that is hosted at Genoscope for analysis. The genome data was integrated to PkGDB database of MicroScope and we have used the gene locus tags from the same database in this study. The genome sequencing data had been submitted to NCBI under accession number PRJNA157903/AKAU00000000.

### Pan–Core Genome Computation

The pan–core genome analysis was computed based on single-linkage clustering algorithm in the software package siLix (SIngle LInkage Clustering of Sequences), hosted by MicroScope. The algorithm implemented in the siLix is based on a principle where a sequence A if considered homologous to sequence B and sequence B is homologous to C, then all the sequences (A, B, and C) are clustered in a same family, irrespective of the similarity between sequences A and C (Miele et al. 2011). Therefore if a gene X is homologous to gene Y, they are clustered together and if Y is a homolog of Z, then all three of them are grouped into the same MicroScope gene family (MICFAM).

### Synteny Calculations

We employed MicroScope platform to compute synteny of *B. terrae* BS001 using data from PkGDB and NCBI RefSeq databases. Genes that satisfied BLASTP alignment threshold (35% sequence identity over 80% length of the smallest protein) or bidirectional best hit criteria, were defined as putative orthologs. Based on these relationships, synteny groups or syntons among other bacterial genomes were searched. The approach allows chromosomal rearrangements including insertions, deletions, and inversions. Gap parameter was set to five genes, representing maximum number of consecutive genes not part of synteny group.

### Bioinformatics and Comparative Genome Analysis

For comparative analysis of genomes, their metabolic profile prediction and pan–core genome analysis and genomic islands predictions, we used MicroScope platform of the Genoscope (http://www.genoscope.cns.fr/agc/microscope/home/index.php, last accessed June 26, 2014). The RGP finder was used to detect more than 5-kb synteny breaks across a query genome and closely related (comparator) ones, followed by screening of the identified region for HGT features, such as the presence of mobility genes, tRNA hot spots and deviation of the G+C% from the main value. It then employed “SIGI-HMM” (based on the use of Hidden Markov Models, using codon usage to characterize the origin of genes) to measure codon usage aberrations (Waack et al. 2006) and “AlienHunter,” which is an “Interpolated Variable Order Motif” that uses variable-order motif distributions (2–8) to exploit compositional biases (Vernikos and Parkhill 2006).

Membrane transporters prediction and annotation were performed using TransAPP (Transporter Automatic Annotation Pipeline) of TransportDB (www.membranetransport.org, last accessed June 26, 2014). Potential type III effectors were predicted using www.effectors.org (last accessed June 26, 2014) with parameters (classification: [standard set] cutoff: [0.9999; selective]), whereas signal peptides were predicted using online server SignalP 4.1 (Petersen et al. 2011). Antibiotics and secondary metabolites analysis shell (antiSMASH) (Blin et al. 2013) online server was used for prediction and analysis of secondary metabolites.

### Metabolic Pathways Comparison

The metabolic profiles of *B. terrae* BS001 and other genomes in this study were analyzed using MicroCyc based on MetaCyc (Caspi et al. 2010) of the MicroScope platform, as a reference pathway database. For each genome, comparative analysis of metabolic pathways was accomplished using MicroScope platform (https://www.genoscope.cns.fr/agc/microscope/metabolism, last accessed June 26, 2014). Each pathway has its own completion value (completion value = the number of enzymatic reactions in a particular pathway in a given organism/the total number of enzymatic reactions in the same pathway in MetaCyc). Hierarchical cluster analysis of the MicroCyc predicted metabolic pathways for *B. terrae* BS001 was performed using MeV tool integrated in the MicroScope, taking result comparisons as input and using pathway completion values. The analysis was performed taking into consideration other *Burkholderia* and a number of additional bacterial genomes integrated in the PkGDB database.

### Phylogenetic Analysis

Phylogenetic trees were made with MEGA 6 (Tamura et al. 2013). Poorly aligned regions and gaps were manually eliminated. Maximum-likelihood method based on the JTT matrix-based model (Jones et al. 1992) was used to infer the evolutionary history for the T3SS, T4SS, ACC-deaminase, and tyrosinase genes. For modeling of evolutionary rate differences among sites discrete Gamma distribution was used (4 categories [+G, parameter = 3.8674]). For network analysis of membrane transporters, amino acid sequences of random ORFs (ATP binding component of ABC and MFS transporters) were aligned using ClustalW in MEGA. The matrix was then analyzed in SplitsTree4 software using WAG model with Gamma 4.0 and ProteinMLdist (characters).

## Supplementary Material

Supplementary figures S1–S5 and tables S1–S10 are available at *Genome Biology and Evolution* online (http://www.gbe.oxfordjournals.org/).

Supplementary Data
