# Supplementary material for: The Genome of the Fungal-Interactive Soil Bacterium Burkholderia terrae BS001—A Plethora of Outstanding Interactive Capabilities Unveiled
Source: Genome Biol Evol. 2014 Jun 12;6(7):1652–68. doi: 10.1093/gbe/evu126 (PMC4122924; doi:10.1093/gbe/evu126)
Supplement: Supplementary Data [file supp_6_7_1652__index.html]

The genome of the fungal-interactive soil bacterium Burkholderia terrae BS001 - A plethora of outstanding interactive capabilities unveiled — The Genome of the Fungal-Interactive Soil Bacterium Burkholderia terrae BS001—A Plethora of Outstanding Interactive Capabilities Unveiled — Supplementary Data 

# The Genome of the Fungal-Interactive Soil Bacterium *Burkholderia terrae* BS001—A Plethora of Outstanding Interactive Capabilities Unveiled

## Supplementary Data

files

**Files in this Data Supplement:**

- Supplementary Data - pdf file
- Supplementary Data - pdf file
- Supplementary Data - xlsx file
- Supplementary Data - tif file
- Supplementary Data - tif file
- Supplementary Data - tif file
