# Supplementary material for: The Genome of the Fungal-Interactive Soil Bacterium Burkholderia terrae BS001—A Plethora of Outstanding Interactive Capabilities Unveiled
Source: Genome Biol Evol. 2014 Jun 12;6(7):1652–68. doi: 10.1093/gbe/evu126 (PMC4122924; doi:10.1093/gbe/evu126)

T6SS cluster 1: (AKAUv1_650058-AKAUv1_650074)

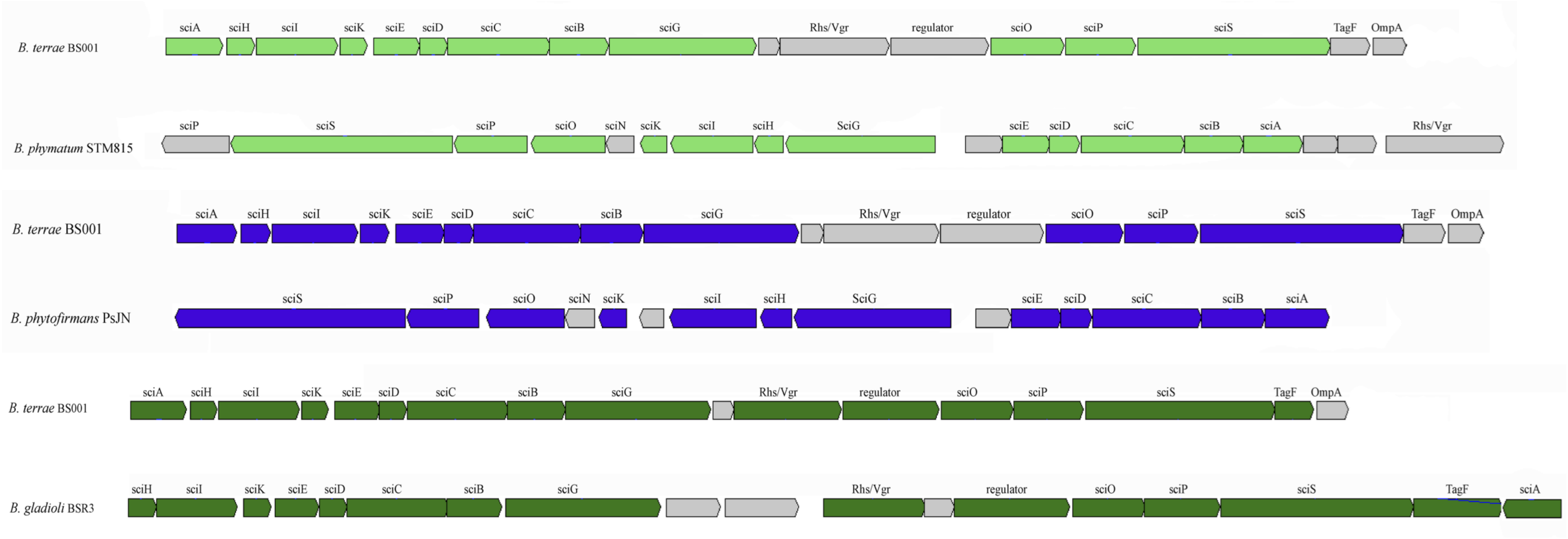

T6SS cluster 2: (AKAUv1_1080195-AKAUv1_1080214)

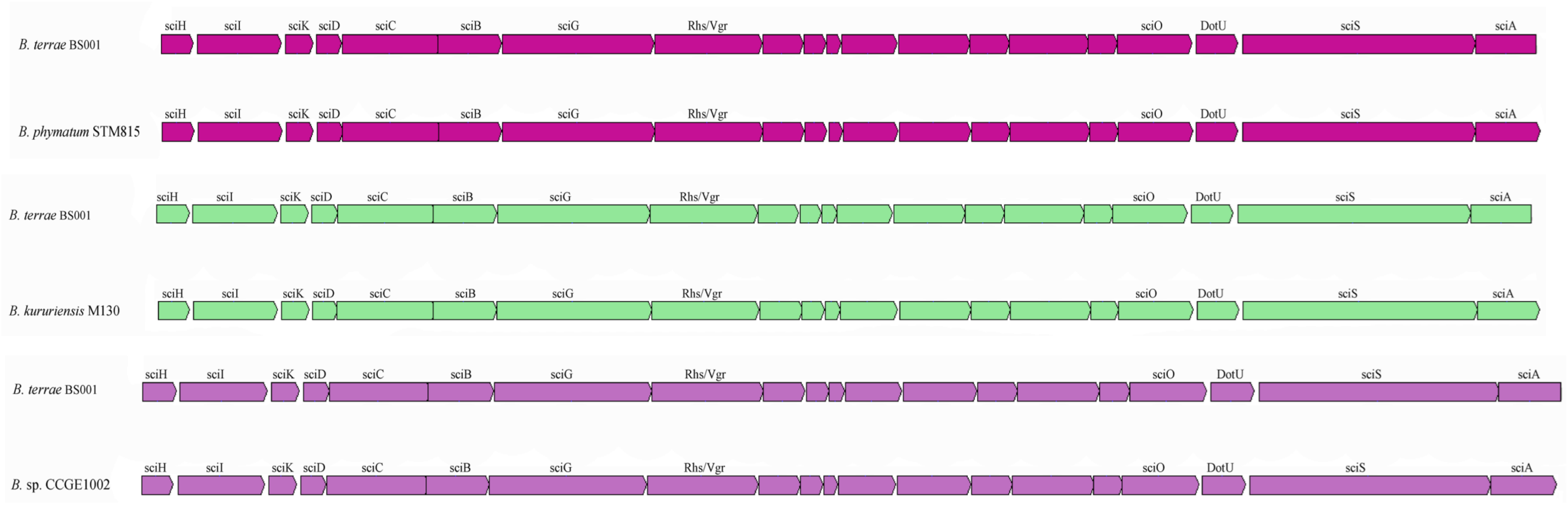

T6SS cluster 3: (AKAUv1_1680089-AKAUv1_1680097)

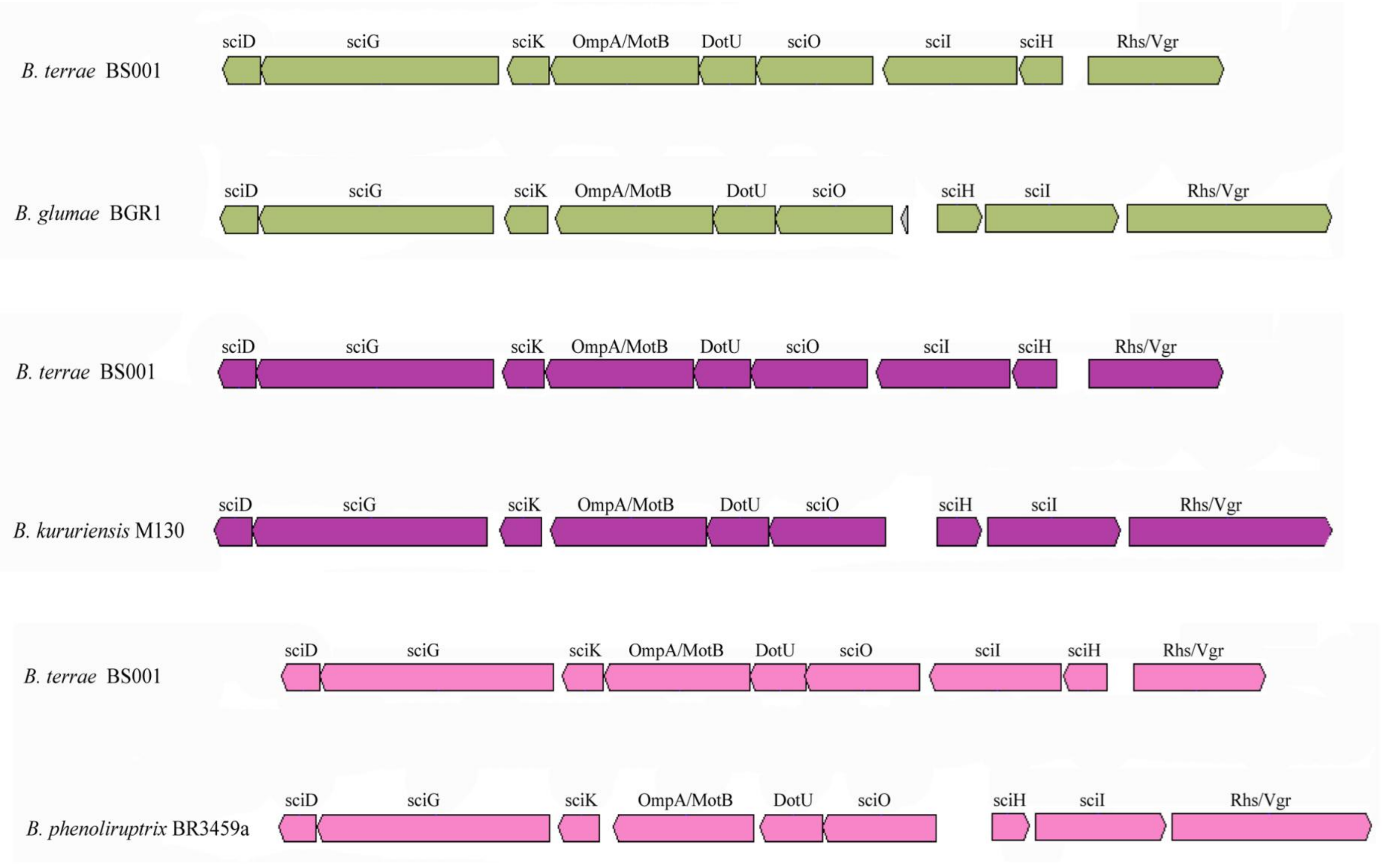

T6SS cluster 4: (AKAUv1_920001-AKAUv1_920005)

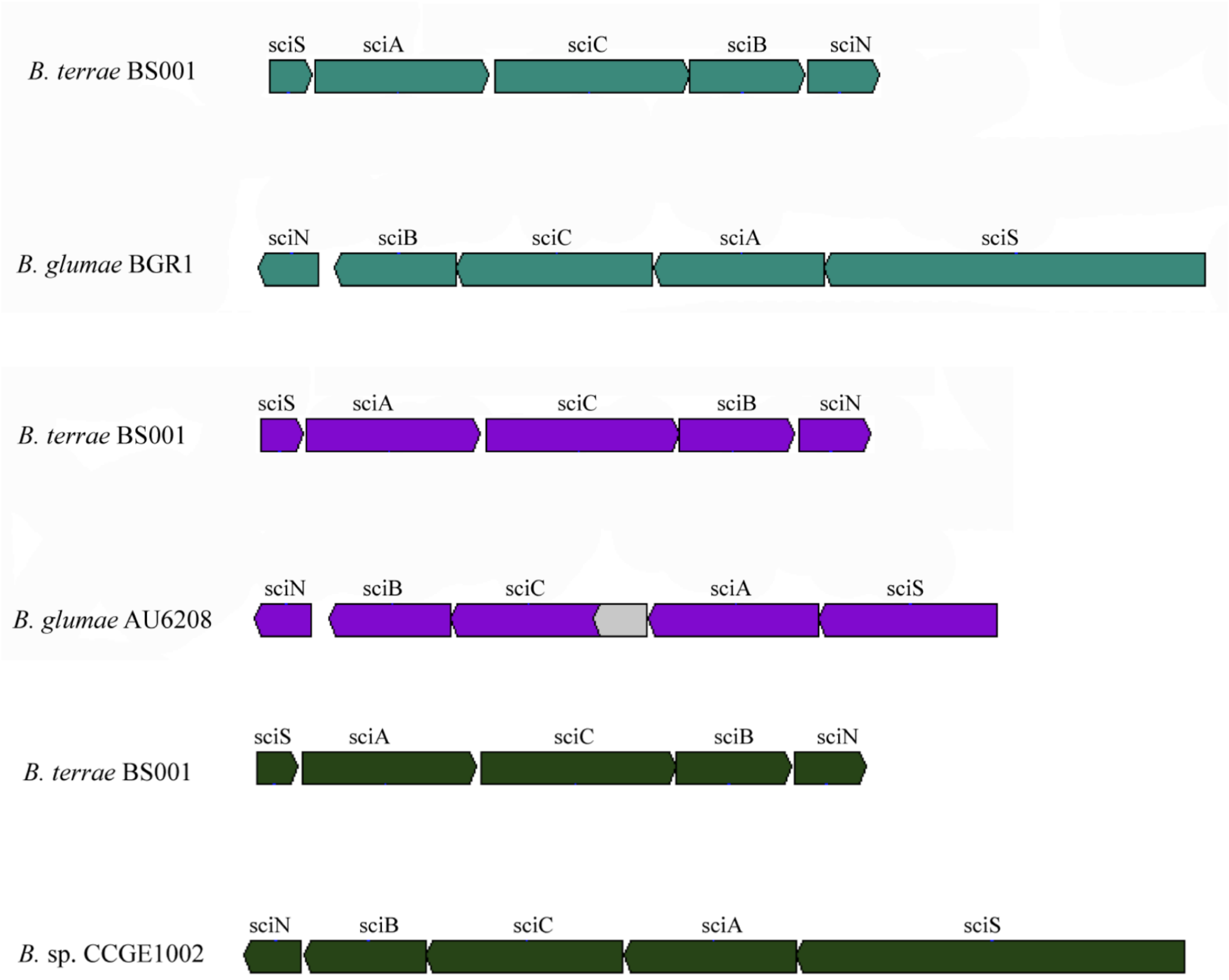

Supplement: Supplementary Data [file supp_evu126_Supplementary_Figure_S2.pdf]
