# Supplementary figures and images for: The Genome of the Fungal-Interactive Soil Bacterium Burkholderia terrae BS001—A Plethora of Outstanding Interactive Capabilities Unveiled
Source: Genome Biol Evol. 2014 Jun 12;6(7):1652–68. doi: 10.1093/gbe/evu126 (PMC4122924; doi:10.1093/gbe/evu126)

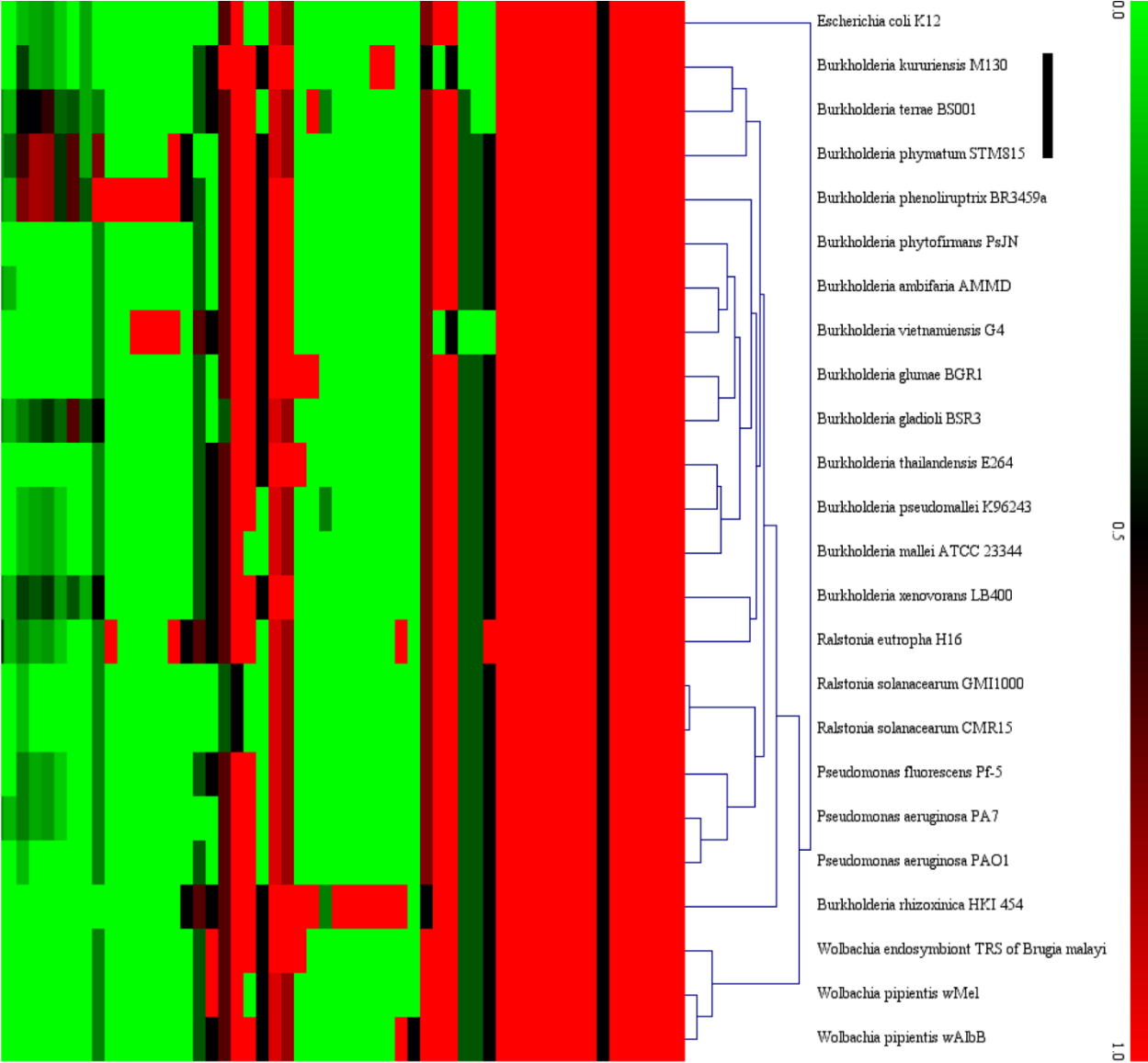

Supplement: Supplementary Data [file supp_evu126_Supplementary_Figure_S3.pdf]

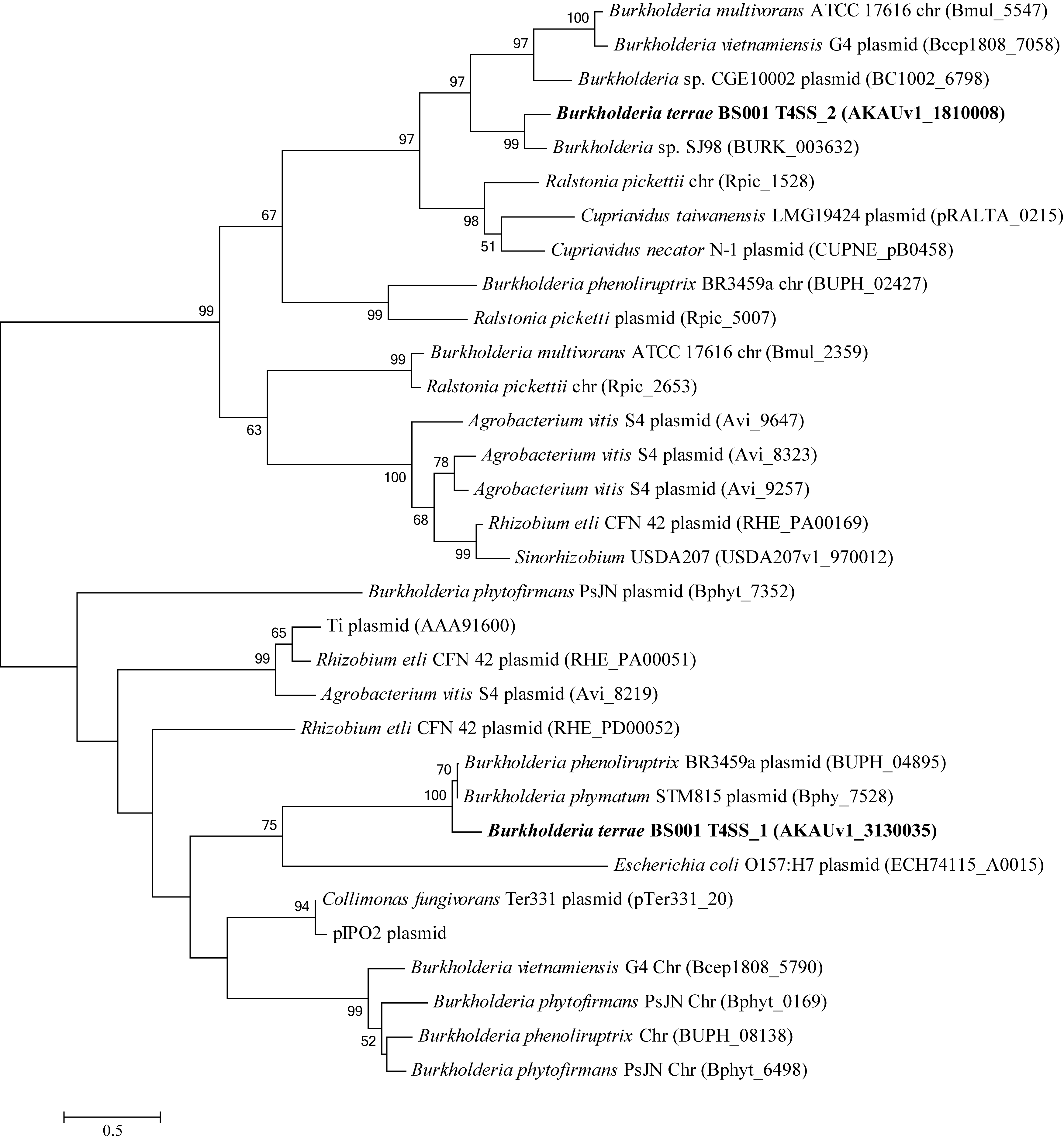

Supplement: Supplementary Data [file supp_evu126_Supplementary_Figure_S1.tif]

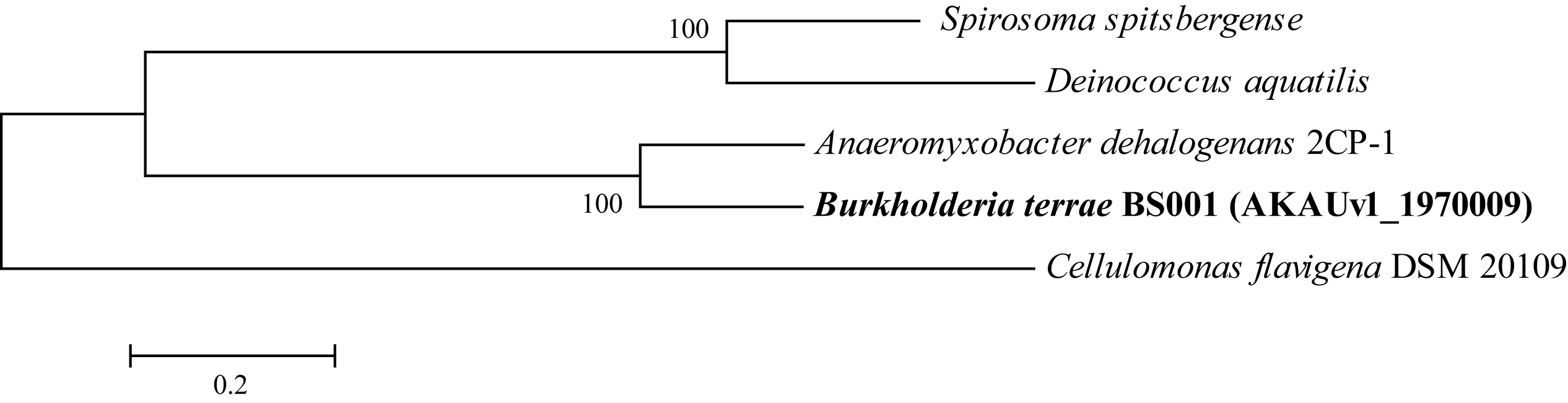

Supplement: Supplementary Data [file supp_evu126_Supplementary_Figure_S4.tif]

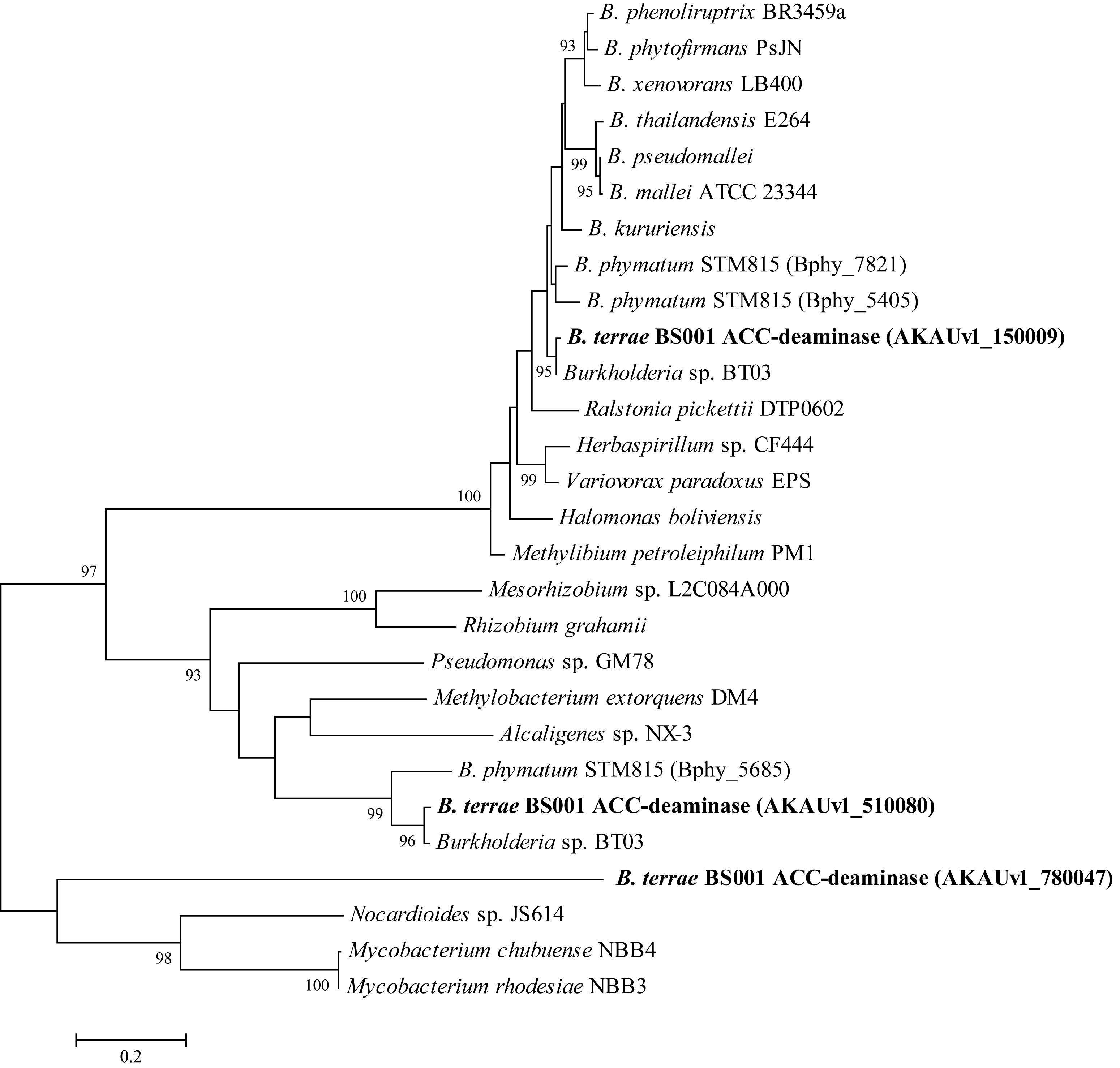

Supplement: Supplementary Data [file supp_evu126_Supplementary_Figure_S5.tif]
